# Comparative analysis of codon usage between *Gossypium hirsutum* and *G. barbadense* mitochondrial genomes

**DOI:** 10.1080/23802359.2020.1780969

**Published:** 2020-06-17

**Authors:** Zhiwen Chen, Jianguo Zhao, Jun Qiao, Weijia Li, Jingwei Li, Ran Xu, Haiyan Wang, Zehui Liu, Baoyan Xing, Jonathan F. Wendel, Corrinne E. Grover

**Affiliations:** a Institute of Carbon Materials Science, Shanxi Datong University, Datong, China; b College of Chemistry and Environment Engineering, Shanxi Datong University, Datong, China; c Department of Ecology, Evolution and Organismal Biology, Iowa State University, Ames, IA, USA

**Keywords:** Gossypium mitochondria, ENC and GC content, preferred codons, neutral mutation, selection

## Abstract

*Gossypium hirsutum* and *G. barbadense* mitochondrial genomes were analyzed to understand the factors shaping codon usage. While most analyses of codon usage suggest minimal to no bias, nucleotide composition, specifically GC content, was significantly correlated with codon usage. In general, both mitochondrial genomes favor codons that end in A or U, with a secondary preference for pyrimidine rich codons. These observations are similar to previous reports of codon usage in cotton nuclear genomes, possibly suggestive of a general bias spanning genomic compartment. Although evidence for codon usage bias is weak for most genes, we identified six genes (i.e. *atp8*, *atp9*, *sdh3*, *sdh4*, *mttB* and *rpl2*) with significant nonrandom codon usage. In general, we find multiple factors that influence cotton mitochondrial genome codon usage, which may include selection in a subset of genes.

## Introduction

Codon usage bias is a common phenomenon in most prokaryotic and eukaryotic species. Biased codon usage can arise as a result of neutral mutations (Osawa et al. 1988; Kano et al. 1991; Liu et al. 2015) or selective constraints (Akashi 1994), and may be different among species or genes within species (Uddin et al. 2018; Bhattacharyya et al. 2019).

Trends in codon usage biases within and among species can provide insight into the different evolutionary pressures that affect genes and species. While there are two main theories at present (i.e. the neutral mutation theory and the selection-mutation-drift theory (Bulmer 1991)), studies have shown that biased codon usage is not determined by a single factor, but may be influenced by factors such as synonymous substitution rates (Sharp and Li 1987; Shi et al. 2006), tRNA abundance (Ikemura 1985; Wei et al. 2019), accuracy of translation (Marais and Duret 2001; Frumkin et al. 2018; Yang et al. 2019), DNA replication sites (Sharp 1991; Sirihongthong et al. 2019), gene length (Moriyama and Powell 1998; Ingvarsson 2007) and expression level (Duret and Mouchiroud 1999; Victor et al. 2019; Liu et al. 2020). In plants, research on codon usage bias has largely focused on nuclear genes, generally finding associations between codon usage and gene function (Chiapello et al. 1998; Liu et al. 2005), some codon usages among species were broadly conserved (Kawabe and Miyashita 2003); however, few studies have characterized codon usage among genes with organelles, such as the mitochondria (Liu et al. 2004; Zhang et al. 2007; Zhou and Li 2009).

Because evolution affects organelles differently, trends in codon usage may be different and/or exaggerated as compared to the nuclear genome (Smith et al. 2012). Genetic drift is usually higher and mutational load tends to be particularly high in the mitochondrial (versus nuclear) genome (Xiao et al. 2017), where more frequent replication and an oxidative environment (due to the reactions that occur within the organelle) can lead to more frequent mutations (Muftuoglu et al. 2014), although this is not broadly observed in angiosperms (Drouin et al. 2008). Studies have shown that mitochondrial, chloroplast, and nuclear genes can exhibit differences in codon usage and evolutionary constraints, as observed in *Triticum aestivum* (Zhang et al. 2007), and natural selection might also play a major role in shaping codon usage bias in plant mitochondrial genomes (Zhou and Li 2009).

Here we report a detailed comparative analysis of codon usage bias in two *Gossypium* mitochondrial genomes using multivariate statistical analysis and correlation analysis to better understand codon usage and evolution in the mitochondrion. We explore the key factors in shaping codon choice in the cotton mitochondrial genomes and provide evidence for translationally optimal codons.

## Materials and methods

### Dataset

The complete mitochondrial genome sequences of *Gossypium hirsutum* and *G. barbadense* were stored in the NCBI database under Genbank Accession Numbers JX065074 and KP898249 (Liu et al. 2013; Tang et al. 2015). 72 CDS sequences, which contained the correct initiation and termination codons from both mitochondrial genomes were used in the analysis. ATG and TGG are unique codons for methionine and tryptophan, which cannot be biased and are excluded.

### Measures of synonymous codon usage bias

‘Relative synonymous codon usage’ (RSCU) (Sharp and Li 1986; Sharp et al. 1986) was calculated for each gene, which is an index to normalize codon usage. RSCU is equivalent to the actual proportional usage of a given codon divided by the expected proportional usage, if all available codons are used equally. If RSCU = 1, codon usage is unbiased. If RSCU > 1, the *i*th codon is used more frequently than other synonymous codons; if RSCU < 1, the *i*th codon is used less frequently.

### Neutrality plot analysis

We quantified the GC content at GC12 (mean content for GC1 and GC2) and GC3 for all codons of an amino acid sequence (excluding Met, Trp, and stop codons), and an a mean GC content for each entire gene. Neutrality plots generated in R.3.2 (https://www.R-project.org/) (R code for all analyses is provided in Supplementary Table S1).

### ENC-plot

The ‘Effective Number of Codons’ (ENC) value can be plotted against the bias in GC to generate an *ENC*-plot. The expected ENC under the null hypothesis (i.e. no selection) was calculated for each gene from the GC3 values, as in Wright (1990):

$$\text{ENC}=2+\text{GC}3+\frac{29}{\text{GC}3^{2}+(1-\text{GC}3)}$$

The ENC ratio, which excludes the effects of different GC content due to neutral mutation, is then estimated as (ENCexp-ENCobs)/ENCexp (Kawabe and Miyashita 2003). The ENC-plots were generated in R.3.2 (https://www.R-project.org/) (R code in Supplementary Table S1).

### Correspondence analysis

Correspondence analysis provides a method for graphically displaying of exploring the relationship between variables in one contingency table. The computational details and rationale of correspondence analysis (CA) were adapted from Greenacre (1993, 2010). Because codon usage patterns and biases can vary among genes, describing codon usage bias for an entire genomic compartment is a multivariate problem. CodonW version 1.4 has an integrated codon bias and correspondence analysis (COA) program designed to evaluate codon usage bias through multivariate statistical analysis (Neron et al. 2009), concomitantly analyzing codon usage across many genes. Here we used CodonW to evaluate the 72 mitochondrial genes (i.e. 36 each in *G. hirsutum* and *G. barbadense*) for evidence of codon usage bias using default parameters. (R code is listed in Supplementary Table S1).

### Determination of optimal codons

Optimal codons were defined as those codons which occurred more often (relative to their synonyms) in highly expressed genes than in lowly expressed genes (Ikemura 1985). The number of genes in each group was 10% of the total number of genes in the dataset. The codon bias of the total codon usage from both sets of genes was estimated using ENC and the set of genes with the lower ENC (more highly biased) was putatively identified as the more highly expressed (Huang et al. 2019). Significance was assessed by a two way chi-squared contingency test with the criterion of 0.01.

### PR2-bias plot analysis

An extension of the base-pair rule (BPR) is the ‘intrastrand parity rule 2’ (PR2), which assumes that the proportion of A = T and G = C should be equivalent *within strand* if there are no biases in mutation, selection, or codon usage to distort these ratios (Sueoka 1995). We calculated PR2-bias plots to evaluate codon usage bias using only third codon positions in those amino acids with four possible codons (Sueoka 1999), i.e. threonine, proline, arginine, leucine, alanine, glycine, valine, and serine. In the present study, ‘A3/(A3 + T3)|4’ and ‘G3/(G3 + C3)|4’ were plotted as the ordinate and abscissa, respectively. A vector from the center represents the extent and direction of the PR2-bias. The PR2-bias plots were plotted in R.3.2 (https://www.R-project.org/) (R code listed in Supplementary Table S1).

## Results

### Analyses reveal unequal nucleotide composition suggestive of codon usage bias

**Figure 1.**
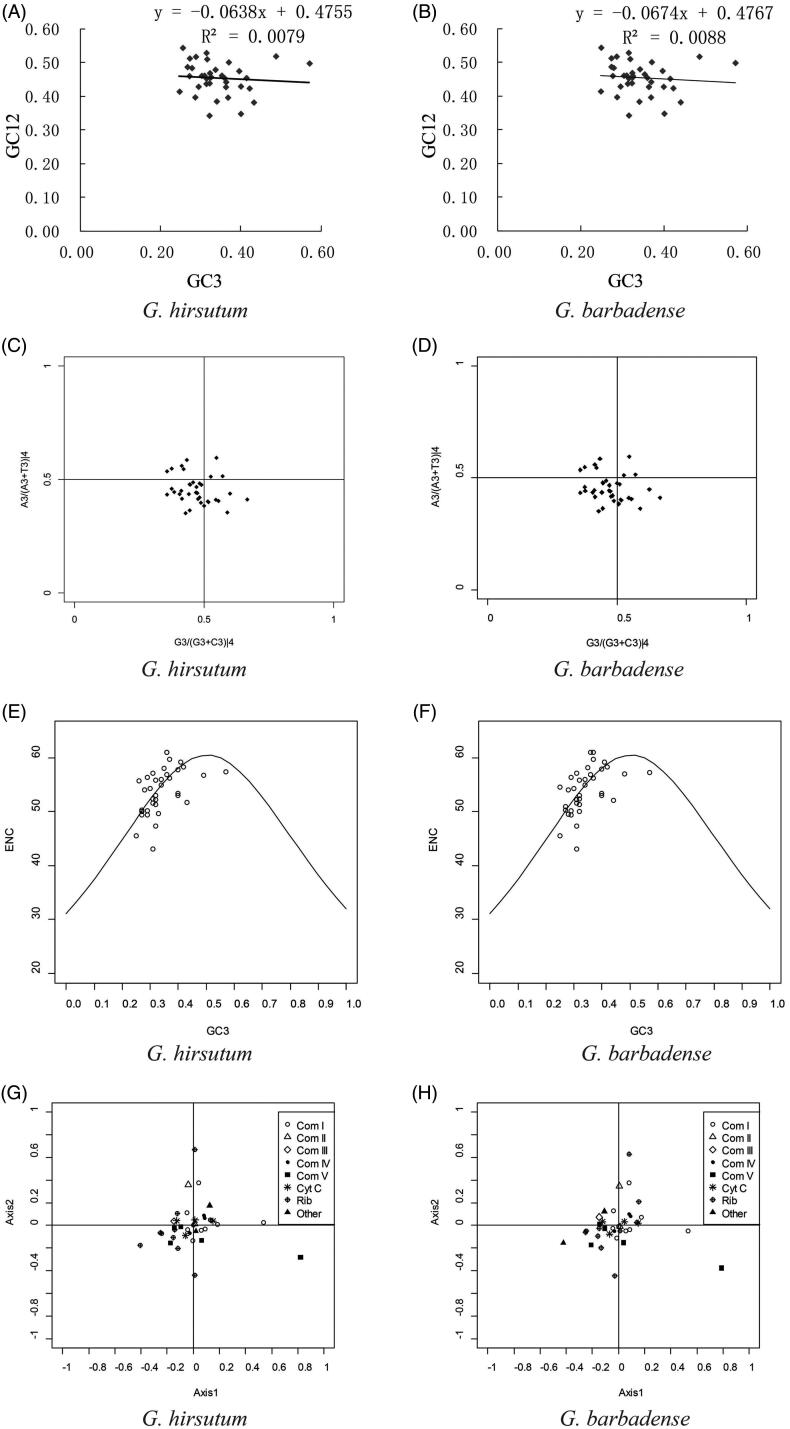
Analysis of codon usage between *G. hirsutum* and *G. barbadense* mitochondrial genes. Neutrality plot analysis (GC3 vs. GC12) of *G. hirsutum* (A) and *G. barbadense* (B). PR2-bias plot of the third codon position for fourfold degenerate amino acids in *G. hirsutum* (C) and *G. barbadense* (D). Each dot in the figure indicates one gene. ENC-plot of the *G. hirsutum* (E) and *G. barbadense* (F) mitochondrial genes. Dots indicate the position of individual genes, and the standard curve represents the expected ENC under random codon usage. Visualization of the first two axes from the correspondence analysis based on RSCU values for *G. hirsutum* (G) and *G. barbadense* (H) mitochondrial genes.

Codon usage biases can be revealed by several different measures, including by comparing the GC content of third codon positions to the first two. Here, we analyzed GC content for different codon positions in *G*. *hirsutum* and *G. barbadense* mitochondrial genes (Supplementary Table S2). Differences in GC content were greatest at the third codon position, which is typically considered neutral with respect to mutations. We generated GC12 vs GC3 neutrality plots for both *G*. *hirsutum* (Figure 1(A)) and *G. barbadense* (Figure 1(B)) using the mitochondrial genes. Notably, most genes are not distributed along the diagonal, reflecting the wide range of GC3 (24.73%–57.10%) and the comparatively relatively narrow range in GC12 distributions (34.27%–54.38%; Supplementary Table S2) and possibly indicating biased codon usage. The correlation coefficient between GC12 and GC3 was 0.0079 in *G*. *hirsutum* and 0.0088 in *G. barbadense*, respectively, neither of which were significant. Likewise, the slope of the regression curve was –0.0638 in *G*. *hirsutum* and –0.0674 in *G. barbadense*, indicating that the correlation between GC12 and GC3 was very weak in both. These results suggest an uneven distribution in mutation between GC12 and GC3 that may suggest a role for selection in generating codon usage bias for these genes.

The intrastrand parity rule 2 (PR2) can further dissect nucleotide biases by plotting the association between purines (A and G) and pyrimidines (C and T) in fourfold (third) degenerate codon positions. We evaluated the *G. hirsutum* and *G. barbadense* genomes for biases in nucleotide usage (Figure 1(C,D)). Most of the mitochondrial genes (20/36 in *G. hirsutum* and 18/36 in *G. barbadense*) were distributed in the third quadrant of the plot, indicating that pyrimidines (C and T) were used more frequently than purines (A and G) at the third codon position, further suggesting a bias in nucleotide usage in cotton mitochondrial genes that potentially reflects non-neutral evolution. Given the general A/T bias in the mitochondrial genes and the slight bias toward pyrimidines, there may exist a slight bias toward codons which end in T for cotton mitochondrial genes.

### Measures of codon usage bias

The idea of ‘Effective Number of Codons,’ or ENC, is a measure of codon usage bias introduced by Wright (1990) to quantify the actual number of codons used by a given gene. The value of ENC, which ranges from 20 to 61, therefore reflects the strength of codon usage bias (complete bias to no bias). The ENC values of the 72 genes in *G. hirsutum* and *G. barbadense* mitochondrial genomes were similar between species, ranging from approximately 43 (*atp9*) to 61 (*atp4* and *rps7*), the latter of which suggests a complete lack of bias. In general, the cotton genes surveyed exhibited higher ENC values (approaching 61; Supplementary Table S2). Because ENC > 35 is not considered strongly biased (Wright 1990; Jiang et al. 2008), the ENC analysis does not suggest strong preferential codon usage for any genes in either cotton species; however, several genes (i.e. *atp9, nad4L, sdh3, cox2, atp6, ccmC*) exhibit ENC < 50, which may be weakly biased.

Because ENC can be influenced by amino acid composition, Wright (1990) outlined a method to calculate the expected ENC values (under purely neutral mutation) which is plotted against GC3 to form a standard curve. Genes whose ENC-GC3 values are distributed along or adjacent to the standard curve may be considered neutrally evolving, whereas those that fall far below the standard curve may be subject to selection (Kawabe and Miyashita 2003). Figure 1(E,F) shows compares the observed values of ENC-GC3 (dots) for cotton mitochondrial genes relative to the expected relationship between ENC and GC3 under H0 (no selection; solid line). As expected from the above analyses, both *G. hirsutum* (Figure 1E) and *G. barbadense* (Figure 1(F)) exhibit nearly identical distributions, many of which were on or near to the standard curve (indicating neutral evolution). Some genes (*atp8*, *atp9*, *sdh3*, *sdh4*, *mttB* and *rpl2*) exhibited ENC values lower than expected, which may be suggestive of other processes influencing nucleotide composition/codon usage, including selection (Kawabe and Miyashita 2003). These results are reiterated in the frequency plots for both species using the ENC ratio ((ENCexp–ENCobs)/ENCexp; Supplementary Figure S1), whereby most genes fall in the ENC ratio bin of 0.0–0.05 (nearly neutral), although the ENC ratio ranges from –0.15 to 0.2, suggesting some genes have an ENC far outside of what is expected, potentially due to non-neutral processes.

A second measure of codon usage bias, i.e. ‘relative synonymous codon usage’ (RSCU), was also used to evaluate codon usage in a normalized fashion. RSCU is an index that is normalized with respect to amino acid composition, thereby permitting a more even comparison among genes or species. The total codon numbers and average RSCU values were listed in Table 1. Seven codons including UUU (Phe), AUU (Ile), UAU (Tyr), GAU(Asp), GAA(Glu), GCU (Ala) and GGU(Gly) were determined as the ‘optimal codons’ (*p* < .01). Among them, the codon GGU(Gly), its usage was significantly more often in highly than in lowly biased genes. Although not considered as the optimal codons, other six codons including UUA (Leu), CAU (His), AAU (Asn), AAA(Lys), CCA (Pro) and CGU (Arg) were used more frequently in the highly expressed dataset at 0.01 < *p* < .05. We found that all of these 13 codons were A/U ended. Congruent with the composition analyses, most of the preferred codons have A- or U- in the third codon position. Although, all the preferred codons ended with A/U, codons with U in the third position were detected more frequently, indicating a general preference for these codons in both cotton mitochondrial genomes.

**Table 1.** Codon usage of high/low expressed gene groups in *G. hirsutum* and *G. barbadense* mitochondrial genomes.

Amino acids	Codon		High		Low	
	G. hirsutum	G. barbadense	G. hirsutum RSCU (No.)	G. barbadense RSCU (No.)	G. hirsutum RSCU (No.)	G. barbadense RSCU (No.)
Phe	UUU*	UUU*	1.35 (38)	1.36 (38)	0.79 (36)	0.80 (36)
	UUC	UUC	0.65 (18)	0.64 (18)	1.21 (54)	1.20 (54)
Leu	UUA@	UUA@	1.51 (25)	1.43 (25)	0.84 (22)	0.85 (22)
	UUG	UUG	1.51 (24)	1.37 (24)	1.26 (32)	1.24 (32)
	CUU	CUU	1.07 (21)	1.20 (21)	1.22 (32)	1.24 (32)
	CUC	CUC	0.39 (9)	0.51 (9)	0.99 (25)	0.97 (25)
	CUA	CUA	1.18 (21)	1.20 (21)	0.88 (23)	0.89 (23)
	CUG	CUG	0.34 (5)	0.29 (5)	0.80 (21)	0.81 (21)
Ile	AUU*	AUU*	1.76 (40)	1.71 (40)	0.81 (40)	0.81 (40)
	AUC	AUC	0.69 (18)	0.77 (18)	1.05 (53)	1.07 (53)
	AUA	AUA	0.56 (12)	0.51 (12)	1.14 (56)	1.13 (56)
Met	AUG	AUG	0.00 (0)	0.00 (0)	0.00 (0)	0.00 (0)
Val	GUU	GUU	1.08 (17)	1.17 (17)	0.70 (17)	0.70 (17)
	GUC	GUC	0.95 (12)	0.83 (12)	1.15 (28)	1.15 (28)
	GUA	GUA	1.29 (19)	1.31 (19)	1.48 (36)	1.48 (36)
	GUG	GUG	0.68 (10)	0.69 (10)	0.66 (16)	0.66 (16)
Tyr	UAU*	UAU*	1.83 (24)	1.85 (24)	1.12 (27)	1.12 (27)
	UAC	UAC	0.17 (2)	0.15 (2)	0.88 (21)	0.88 (21)
TER	UAA	UAA	0.00 (0)	0.00 (0)	0.00 (0)	0.00 (0)
	UAG	UAG	0.00 (0)	0.00 (0)	0.00 (0)	0.00 (0)
His	CAU@	CAU@	1.83 (8)	1.78 (8)	1.07 (23)	1.07 (23)
	CAC	CAC	0.17 (1)	0.22 (1)	0.93 (20)	0.93 (20)
Gln	CAA	CAA	1.64 (19)	1.73 (19)	1.43 (41)	1.44 (41)
	CAG	CAG	0.36 (3)	0.27 (3)	0.57 (16)	0.56 (16)
Asn	AAU@	AAU@	1.55 (16)	1.52 (16)	1.00 (32)	1.00 (32)
	AAC	AAC	0.45 (5)	0.48 (5)	1.00 (32)	1.00 (32)
Lys	AAA@	AAA@	1.38 (22)	1.47 (22)	1.03 (77)	1.03 (77)
	AAG	AAG	0.62 (8)	0.53 (8)	0.97 (73)	0.97 (73)
Asp	GAU*	GAU*	1.80 (15)	1.88 (15)	0.94 (32)	0.94 (32)
	GAC	GAC	0.20 (1)	0.12 (1)	1.06 (36)	1.06 (36)
Glu	GAA*	GAA*	1.79 (24)	1.78 (24)	1.13 (53)	1.13 (53)
	GAG	GAG	0.21 (3)	0.22 (3)	0.87 (41)	0.87 (41)
Ser	UCU	UCU	1.66 (22)	1.52 (22)	1.15 (31)	1.14 (31)
	UCC	UCC	0.65 (9)	0.62 (9)	1.07 (29)	1.07 (29)
	UCA	UCA	1.16 (16)	1.10 (16)	0.81 (22)	0.81 (22)
	UCG	UCG	1.23 (18)	1.24 (18)	1.07 (29)	1.07 (29)
Pro	CCU	CCU	1.12 (17)	1.24 (17)	0.92 (21)	0.92 (21)
	CCC	CCC	0.77 (9)	0.65 (9)	1.27 (29)	1.27 (29)
	CCA@	CCA@	1.40 (21)	1.53 (21)	0.88 (20)	0.88 (20)
	CCG	CCG	0.70 (8)	0.58 (8)	0.92 (21)	0.92 (21)
Thr	ACU	ACU	1.10 (8)	0.94 (8)	1.04 (22)	1.04 (22)
	ACC	ACC	0.69 (7)	0.82 (7)	1.22 (26)	1.22 (26)
	ACA	ACA	1.52 (12)	1.41 (12)	0.85 (18)	0.85 (18)
	ACG	ACG	0.69 (7)	0.82 (7)	0.89 (19)	0.89 (19)
Ala	GCU*	GCU*	2.13 (34)	2.00 (34)	1.11 (30)	1.11 (30)
	GCC	GCC	0.67 (12)	0.71 (12)	1.22 (33)	1.22 (33)
	GCA	GCA	0.87 (13)	0.76 (13)	0.74 (20)	0.74 (20)
	GCG	GCG	0.33 (9)	0.53 (9)	0.93 (25)	0.93 (25)
Cys	UGU	UGU	1.45 (9)	1.50 (9)	0.90 (13)	0.90 (13)
	UGC	UGC	0.55 (3)	0.50 (3)	1.10 (16)	1.10 (16)
TER	UGA	UGA	0.00 (0)	0.00 (0)	0.00 (0)	0.00 (0)
Trp	UGG	UGG	0.00 (0)	0.00 (0)	0.00 (0)	0.00 (0)
Arg	CGU@	CGU@	1.43 (13)	1.73 (13)	0.82 (25)	0.82 (25)
	CGC	CGC	0.13 (1)	0.00 (0)	0.65 (20)	0.66 (20)
	CGA	CGA	1.96 (14)	1.87 (14)	1.08 (33)	1.08 (33)
	CGG	CGG	1.17 (8)	1.07 (8)	0.72 (22)	0.72 (22)
Ser	AGU	AGU	0.94 (17)	1.17 (17)	0.67 (18)	0.66 (18)
	AGC	AGC	0.36 (5)	0.34 (5)	1.22 (34)	1.25 (34)
Arg	AGA	AGA	1.30 (9)	1.20 (9)	1.53 (46)	1.51 (46)
	AGG	AGG	0.00 (0)	0.13 (1)	1.21 (37)	1.21 (37)
Gly	GGU*	GGU*	2.00 (34)	2.00 (34)	0.97 (32)	0.97 (32)
	GGC	GGC	0.45 (6)	0.35 (6)	0.73 (24)	0.73 (24)
	GGA	GGA	1.23 (21)	1.24 (21)	1.12 (37)	1.12 (37)
	GGG	GGG	0.32 (7)	0.41 (7)	1.18 (39)	1.18 (39)

High bias was assigned to the dataset with the lower average ENC. Optimal codons were indicated with ‘*’ (*p* < .01). Codons that occurred more frequently in the highly expressed dataset at 0.01 < *p* < .05 were indicated with ‘@.’

We further evaluated the RSCU patterns using correspondence analysis (Figure 1(G,H)). The first two axes explain relatively small amounts of the overall variance in codon usage, i.e. 10.16% (*G. hirsutum*) and 10.39% (*G. barbadense*) for Axis 1, and 3.14% (G*. hirsutum*) and 1.97% (*G. barbadense*) for Axis 2, which indicates no strong trends in the data. These results indicate that the interplay between several factors (e.g. neutral mutation, selection, etc) is likely responsible for shaping codon usage in *Gossypium* mitochondrial genome genes. When we further divide the analysis based on the mitochondrial complexes (Figure 1(G,H)), most genes are centrally clustered, others (e.g. ATP synthase from Complex V) are somewhat more dispersed, potentially indicating differences in codon usage within that complex.

## Discussion

The evolutionary significance and mechanisms that underlie biased codon usage have been debated for decades. Although the redundancy in the genetic code suggests complete interchangeability among synonymous codons, biases in codon usage are frequent (Ikemura 1985; Hershberg and Petrov 2008), although not ubiquitous (Duret 2002), and may be consistent (Chen et al. 2004) or variable (Ikemura 1985; Sharp et al. 1988) among genes within a genome. A number of processes can influence codon usage, including neutral mutations, genetic drift, variability in GC content, and selection, among others (Liu et al. 2015; Song et al. 2017; Xiao et al. 2017). Weak patterns of codon usage bias have been reported for cotton nuclear genes (Wang et al. 2018), exhibiting slight preferences for codons that end in A or T and/or pyrimidine rich codons associated with nucleotide composition. Because the mitochondria genome is subject to a related yet unique evolutionary history influenced by different evolutionary factors (Chen et al. 2017), we evaluated the mitochondrial genomes of *G. hirsutum* and *G. barbadense* for evidence of codon usage biases.

Similar to cotton nuclear genes (Wang et al. 2018), codon usage bias in the mitochondrial genome was weak. In general, the codon usage biases suggested here were similarly associated with nucleotide composition. Like cotton nuclear genes, cotton mitochondrial genes tend to be GC-poor in the third codon position, which leads to a slight bias toward codons that end in either A or T. RSCU analyses generally agreed with these results, revealing that the more frequently used codons typically ended with either A or T in the third codon position. Notably, two of the highly preferred codons we detected here (GCU and GGU) were also among the seven and twelve preferred codons detected in the *G. hirsutum* chloroplast (Shang et al. 2011) and nuclear (Wang et al. 2018) genomes, suggesting a general preference for these codons in cotton.

In addition, the results of the codon usage patterns between *Gossypium hirsutum* and *G. barbadense* mitochondrial genomes are basically the same. These findings suggested that the codon usage patterns of *Gossypium* mitochondrial genomes were highly conserved, which indicated that the dominant selection pressure for codon usage appears to be purifying selection and suggested that similar genome-changing mechanisms operated in the long-term evolution of cotton (Guan et al. 2019).

Our results suggested that biased usage toward preferred codons promote the efficiency of transcription and translation, which is an indicator of combined efforts of evolutionary forces (Chaney and Clark 2015; Shah et al. 2015). Based on this view, the codon usage bias observed in cotton could have lower biological functional significance than expected, only to show that they have experienced strong purifying selection (Hershberg and Petrov 2008; Jia and Higgs 2008; James et al. 2016). Thus, to test if the extent of codon usage reflects various biological processes, further studies on transcriptional and translational mechanisms in cotton are required. The expression data of mitochondrial genes between codon usage and expression level in cotton are also required to establish the connection.

## Data Availability

The data that support the findings of this study are openly available in the NCBI database under Genbank Accession Numbers JX065074 (https://www.ncbi.nlm.nih.gov/nuccore/JX065074.1/) and KP898249 (https://www.ncbi.nlm.nih.gov/nuccore/KP898249).
